# Supplementary material for: Comparative analysis of patient-reported outcomes in joint arthroplasty surgeries
Source: PLoS One. 2024 Dec 23;19(12):e0314818. doi: 10.1371/journal.pone.0314818 (PMC11666041; doi:10.1371/journal.pone.0314818)
Supplement: S1 File — (PDF) [file pone.0314818.s001.pdf]

TurkuCRC täyttää:

Lupapäätösnumero	Lupa myönnetty ajalle	Tutkimuksen projektinumero
T01/019/20	2021 -2030	—

1. Tutkimusnumero, (Esim. T1/2015) T283/2020

☒ Uusi tutkimus

☐ Muutos vanhaan tutkimuslupaan. Mitä muutos koskee?

☐ Tutkijan/tutkijoiden lisäys, ilmoitusmenettely. Täytetään kohdat 4, 5, 6 ja 7. Vastuullinen tutkija allekirjoittaa. Toimitetaan TurkuCRC:lle.

2. Tutkimustyyppi

☐ Opinnäytetyö (syventävät opinnot, pro gradu, väitöskirja jne)

☐ Tutkijalähtöinen tutkimus

☐ Toimeksiantajalähtöinen tutkimus, toimeksiantaja:

☐ Ihminen tutkimuksen kohteena

- ☐ lääketutkimus
- ☐ laitetutkimus
- ☐ muu lääketieteellinen tutkimus (kajoava)
- ☐ kysely- tai haastattelututkimus
- ☐ muu tutkimus, jossa ihminen tutkimuksen kohteena

☒ Asiakirja tai rekisteri tutkimuksen kohteena

☐ Kudos tai ihmisperäinen näyte tutkimuksen kohteena

a. näytetutkimus

☐ jossa rekisteröidyiltä pyydetään suostumukset. Kuvaile suostumuksenpyyntöprosessi

☐ jolle haetaan Fimean lupa ja eettisen toimikunnan puoltava lausunto

☐ jossa näytteenantajat ovat kuolleet; ja tutkimukselle haetaan eettisen toimikunnan puoltava lausunto.

☐ jossa ei missään vaiheessa käsitellä henkilötietoja

b. biopankkitutkimus

☐ jossa tietoja tarvitaan biopankin näyte- ja tietorekisterin lisäksi VSSHP:n potilasrekisteristä

☐ jossa kaikki tutkimusmateriaali saadaan biopankin näyte- ja tietorekisteristä (mutta tutkimuksen toteutukseen tarvitaan VSSHP:n tiloja/laitteita)

☐ Muu tieteellinen tukimus

3. Rekisterinpitäjä (Huom. VSSHP ei pääsääntöisesti ole opinnäytetöiden rekisterinpitäjä)

☒ VSSHP:n kuntayhtymä (liitteeksi Tieteellisen tutkimuksen tietosuoja ja vaikutustenarviointi)

☐ Turun yliopisto (liitteeksi Tieteellisen tutkimuksen tietosuoja ja vaikutustenarviointi)

☐ VSSHP:n ja TUrun yliopiston yhteisrekisterinpitäjyys (liitteeksi Tieteellisen tutkimuksen tietosuoja ja vaikutustenarviointi)

☐ Muu (liitteeksi Tieteellisen tutkimuksen tietosuoja ja vaikutustenarviointi tai muu vastaava seloste)

4. Tutkimuksen nimi ja mahdollinen lyhenne
Ortopedisen hoidon laatu ja sen kehittäminen

5. Vastuullinen tutkija (Opinnäytetöissä vastuullinen tutkija on opinnäytetyön ohjaaja)
(Nimi, toimialue, sähköposti, puhelinnumero)
Ville Äärimaa, Tules toimialue
ville.aarimaa@tyks.fi, 050-4633235

Yhteyshenkilö
(Nimi, sähköposti, puhelinnumero)

6. Opinnäytetyön tekijän nimi, oppiarvo/virka, toimipaikka, sähköpostiosoite (täytetään vain opinnäytetöistä)

☐ olen ollut yhteydessä yksikköön, jossa aion opinnäytetyön suorittaa. Yksikkö ja henkilöt:

7. Muut tutkimuksen/ työn tekemiseen VSSHP:ssä osallistuvat henkilöt, joilla on pääsy käytettävään potilaskertomustietoon (kohtien 5-6 henkilöiden lisäksi)
Nimi, oppiarvo/virka, toimipaikka, sähköpostiosoite

Paula Vainikainen, ylihoitaja, Tules, paula.vainikainen@tyks.fi
Suvi Lähteenmäki, tiedotussuunnittelija, Tules, suvi.lahteenmaki@tyks.fi
Mia Aho, suunnittelija, Tules, mia.karoliina.aho@tyks.fi

Lisäksi erillisellä liitteellä (Liite 1) Tules osastonhoitajat ja erikoislääkärit

8. Sisäiset ostopalvelut

☐ Tykslab
☐ Varsinais-Suomen kuvantamiskeskus
☐ Patologia
☐ Sairaala-apteekki
☐ Kliininen neurofysiologia
☐ Tyks mikrobiologia ja genetiikka

KLIP: ☐ Kliininen fysiologia
☐ Isotooppiosasto
☐ PET-keskus

☒ Auria tietopalvelu
☐ Auria biopankki
☐ muu, mikä

9. Kustannukset

☐ Tutkimukselle perustetaan uusi projektinumero

☐ Kustannukset katetaan jo olemassa olevalta projektinumerolta ____________(esim. 17065 tai 13705)

☒ Ei tutkimuksesta aiheutuvia kustannuksia, jotka laskutettaisiin VSSHP:n projektinumeron kautta

10. Lyhyt selvitys toimialueen resurssien käytöstä (tarvittaessa liitteenä)

☒ Käytetään VSSHP:n tiloja tai laitteita. Mitä ja kenen kanssa asiasta on sovittu?
☐ Tarvitaan tutkimukseen kuulumattoman henkilökunnan (esim. sihteerien) työpanosta.
☐ Käytetään VSSHP:n muita resursseja.

Lisää selvitys kaikista valituista kohdista.
Työ tehdään Tules toimialueen tiloissa

11. Tutkimuksen/työn kesto. Mille ajalle tutkimuslupaa haetaan? (lupa myönnetään pääsääntöisesti enintään viideksi vuodeksi kerrallaan, paitsi rekisteritutkimuksissa enintään kymmeneksi vuodeksi)

11/20 - 11/30

12. Tarvittavien tietojen yksilöinti rekisteritutkimuksessa

a. Poimitko tiedot itse potilaskertomuksesta vai tarvitsetko poimintapalvelua (ks. ohje)

☐ poimin itse

☒ tarvitsen poimintapalvelua

b. MIllä kriteereillä potilaat valitaan kohorttiin?
Toimenpiteistä ja diagnooseista numerot. Uusi toimenpideluokitus 1997 alkaen, diagnoosit: ICD-8: 1977-1986, ICD-9: 1987-1995, ICD-10:1996 alkaen

Tuki- ja liikuntaelinsairauksista kärsivät potilaat ICD-koodin ja toimenpidekoodin perusteella poimittuna. Liitteenä (Liite 2) esimerkkilistaus diagnoosikoodeista potilasprosesseittain.

c. Potilasrekisteristä poimittavat tiedot sekä vuodet tai ajanjakso

13. Onko tutkimus rekisteröity julkiseen tutkimusrekisteriin (ClinicalTrials.gov)?

☐ **Kyllä, NCTnumero _____**

☒ **Ei, miksi?** ☒ **kyseessä ei ole interventiotutkimus**

☐ **muu syy, mikä**

14. Lisätietoja
Tutkimuksen tarkoituksena on potilashoidon vaikuttavuuden arviointi ja jatkokehittäminen, tietoaltaan rekisteritietoja ja hoitotulokseen vaikuttavia muuttujia ja toimintatapoja vertailemalla.

Liitteet

Hakemuksen liitteet:

☐ Kustannuserittely (valmis excel-pohja tai vapaamuotoinen)
☒ Tutkimussuunnitelma, **pakollinen liite**
☐ Sisäiset ostopalvelusopimukset
☐ Tutkimus- ja/tai yhteistyösopimukset ja/tai muu rahoituspäätös
☐ Eettisen toimikunnan lausunto/lausunnot
☐ Fimean käsittelyilmoitus
☐ Findatan lupa
☐ Muu viranomainen, mikä
☒ Tieteellisen tutkimuksen tietosuoja ja vaikutustenarviointi tai muu vastaava seloste, ks. kohta 3.
☐ Malli tutkittavan informoimiseksi laadittavasta tiedotteesta ja suostumuksesta
☐ Ulkopuolinen hakija, CV

Vastuullisen tutkijan allekirjoitus (kohdassa 5 ilmoitettu henkilö)

Allekirjoituksellani sitoudun omasta ja tietoja käsittelevän ryhmän puolesta tietojen salassapitoon ja niiden käyttöön vain lupapäätöksen ehtojen mukaisesti.

Sitoudun siihen, että tutkimuksessa noudatetaan hyvää tutkimustapaa ja tieteellistä käytäntöä ja että tutkimuksen tulokset julkaistaan viivyttelemättä riippumatta siitä, ovatko ne hakijalle tai tutkimuksen rahoittajille toivottuja tai ei.

Mahdolliset epäilyt hyvän tieteellisen käytännön loukkaamisesta käsitellään noudattaen Tutkimuseettisen neuvottelukunnan ohjetta "Hyvä tieteellinen käytäntö ja sen loukkausepäilyjen käsitteleminen Suomessa" (www.tenk.fi).

Nimi Ville Äärimaa

Päiväys 5.11.2020

Allekirjoitus [signature]

Lomake toimitetaan liitteineen TurkuCRC:hen (rakennus 9, 2 kerros)
VSSHP, Hallintokeskus
PL 52
20521 Turku
TurkuCRC toimittaa lomakkeen puollettavaksi ja hyväksyttäväksi. Saatte lupapäätöksen sähköpostiinne.

Toimialueen, palvelualueen, tulosalueen tai liikelaitoksen
TUTKIMUKSEN JA OPETUKSEN VASTUUHENKILÖN JA/TAI YLIHOITAJAN PUOLTO

Päätösnumero

Päiväys 15.1.21

Allekirjoitus [signature]

Nimenselvennys Anton Palomäki
ort. & trau oppiaineen vastuuhenkilön sij

Toimialueen, palvelualueen, tulosalueen tai liikelaitoksen johtajan, johtajaylilääkärin, ylihoitajan tai hallintoylihoitajan päätös, VSSHP:n tutkimuslupa

Päätösnumero

Päiväys 18.1.2021

Allekirjoitus [signature]

Nimenselvennys Petri Virolainen
[illegible] sairaalajohtaja

Luvan edellytykset

Lupa tietojen saamiseen salassa pidettävästä asiakirjasta voidaan myöntää hakijalle tieteellistä tutkimusta, tilastointia tai viranomaisen suunnittelu- tai selvitystyötä varten. Lupa voidaan myöntää, jos on ilmeistä, ettei tiedon antaminen loukkaa niitä etuja, joiden suojaksi salassapitovelvollisuus on säädetty.

Luvan ehdot

- Luvan nojalla saadut tiedot ovat salassa pidettäviä ja niitä saa käyttää vain lupahakemuksen liitteenä olevassa tutkimussuunnitelmassa määriteltyyn tutkimukseen.
- Tutkimuksen muut tiedot tulee saada laillisesti joko viranomaisluvalla tai tutkittavan suostumuksella.
- Saatuja tietoja ei saa luovuttaa, siirtää taikka myydä kolmannelle osapuolelle, eikä niitä voida liittää muihin kuin tätä tutkimusta varten suostumuksella tai viranomaisluvalla saatuihin tietoihin tai rekistereihin.
- Tutkimuksen aikana tutkimusrekisterin pitäjän on huolehdittava siitä, että tutkimuksessa muodostuvat yksittäisen henkilön identifioinnin mahdollistavat tutkimusaineistot säilytetään tutkimuksen aikana omina, potilasasiakirjoista / sosiaalihuollon asiakirjoista / muun asiakas-, palvelu- tai hallintotoiminnan asiakirjoista erillisinä aineistoinaan ja suojattuina asiattomalta pääsyltä tietoihin kaikissa käsittelyn vaiheissa sekä manuaalisten että atk-tiedostojen osalta siten, että vain luvassa mainituilla henkilöillä on oikeus käsitellä tietoja.
- Luvan nojalla saatuja salassa pidettäviä tietoja ei käytetä yksittäisiä tutkimushenkilöitä koskevassa päätöksenteossa.
- Tutkimuksen tulokset tulee julkistaa.
- Tietosuojasyistä tutkimuksen tulokset tulee julkaista siten, ettei niistä voi tunnistaa yksittäisiä henkilöitä. Tulosten raportoinnissa ja julkaisemisessa on noudatettava tieteen yleisiä eettisiä ohjeita.
- Tutkimusluvan saajan on toimitettava tutkimusluvan myöntäneelle viranomaiselle julkaisujen kopiot tai muu vastaava selvitys tutkimuksen etenemisestä tutkimuksen päättyessä.
- Tutkimuksen päätyttyä yksittäisen henkilön identifioinnin mahdollistava tutkimusaineisto tulee hävittää tai siirtää arkistoitavaksi tai sen tiedot tulee muuttaa sellaiseen muotoon, ettei tiedon kohde ole niistä tunnistettavissa, kun henkilötiedot eivät ole enää tarpeen tutkimuksen suorittamiseksi tai sen tulosten asianmukaisuuden varmistamiseksi.
- Yksityisen tutkimusrekisterin osalta henkilötietoja sisältävä tutkimusaineisto voidaan arkistoida vain, jos se on tieteellisen tutkimuksen kannalta tai muusta syystä merkityksellinen ja kansallisarkisto on antanut siihen luvan. Aineisto tulee arkistoida korkeakoulun tai tutkimustyötä lakisääteisenä tehtävänä suorittavan laitoksen tai viranomaisen arkistoon kansallisarkiston määräysten mukaisesti. Kansallisarkisto voi antaa yhteisölle, säätiölle ja laitokselle luvan siirtää arkistoonsa omassa toiminnassaan syntyneitä henkilötietoja sisältäviä tutkimusaineistoja, jotka ovat tieteellisesti tai muusta syystä merkittäviä.
- Lupa voidaan peruuttaa, jos lupapäätökseen sisältyviä ehtoja rikotaan, jolloin luvan saajan on palautettava tutkimusta varten saamansa tiedot.
- Tutkimuksen vastuullisen johtajan tulee antaa lupapäätös tiedoksi kaikille tutkimusryhmän jäsenille ja valvoa lupaehtojen noudattamista.
- Jos tutkimusta suorittavassa organisaatiossa tai rekisterinpitäjän osalta tapahtuu olennaisia muutoksia, niistä tulee ilmoittaa luvan myöntäjälle, joka harkitsee edellyttääkö muutos uutta lupaa.

Liite 1: Muut tutkimuksen / työn tekemiseen Vsshp:ssä osallistuvat henkilöt, joilla on pääsy käytettävään potilaskertomustietoon

Tules osastonhoitajat:
Kaisa Suuripää, kaisa.suuripaa@tyks.fi
Vuokko Sahi-Puolakka, vuokko.sahi-puolakka@tyks.fi
Teija Tiusanen, teija.tiusanen@tyks.fi
Annukka Haataja, annukka.haataja@tyks.fi
Elina Lauas, elina.lauas@tyks.fi
Pia Sykkö, pia.sykko@tyks.fi

Tules erikoislääkärit:
Riku Alaranta, riku.alaranta@tyks.fi
Jevgeni Aniskov, jevgeni.aniskov@tyks.fi
Anssi Arimaa, anssi.arimaa@tyks.fi
Hannu Aro, hannu.aro@tyks.fi
Olli Birling, olli.birling@tyks.fi
Elina Ekman, elina.ekman@tyks.fi
Tuuli Erjanti, tuuli.erjanti@tyks.fi
Minna Forsman, minna.forsman@tyks.fi
Juho Hatakka, juho.hatakka@tyks.fi
Juha Helminen, juha.helminen@tyks.fi
Matias Hemmilä, matias.hemmila@tyks.fi
Kari Isotalo, kari.isotalo@tyks.fi
Petteri Jokinen, petteri.jokinen@tyks.fi
Satu Jokinen-Valovirta, satu.jokinen-valovirta@tyks.fi
Juhani Juhola, juhani.juhola@tyks.fi
Mikko Karvonen, mikko.karvonen@tyks.fi
Hannes Keemu, hannes.keemu@tyks.fi
Karri Kirjasuo, karri.kirjasuo@tyks.fi
Jani Knifsund, jani.knifsund@tyks.fi
Kirsi Korpela, kirsi.korpela@tyks.fi
Juha Kukkonen, juha.kukkonen@tyks.fi
Kaisa Lehtimäki, kaisa.lehtimaki@tyks.fi
Tiina Leistevuo, tiina.leistevuo@tyks.fi
Heli Lähdeniemi, heli.lahdeniemi@tyks.fi
Annika Miikkulainen, annika.miikkulainen@tyks.fi
Keijo Mäkelä, keijo.makela@tyks.fi
Petri Paakki, petri.paakki@tyks.fi
Antton Palomäki, antton.palomaki@tyks.fi
Joonatan Pappinen, joonatan.pappinen@tyks.fi
Katri Pernaa, katri.pernaa@tyks.fi
Markus Pääkkönen, markus.paakkonen@tyks.fi
Juho Rantakokko, juho.rantakokko@tyks.fi
Ida Rantalaiho, ida.rantalaiho@tyks.fi
Anssi Ryösä, anssi.ryosa@tyks.fi
Mikhail Saltychev, mikhail.saltychev@tyks.fi
Pjotr Sarantsin, pjotr.sarantsin@tyks.fi
Miika Stenholm, miika.stenholm@tyks.fi
Matti Seppänen, matti.seppanen@tyks.fi
Stefan Suvitie, stefan.suvitie@tyks.fi
Tuukka Tanskanen, tuukka.tanskanen@tyks.fi
Hanna-Stiina Taskinen, hanna-stiina.taskinen@tyks.fi
Petteri Unkuri, petteri.unkuri@tyks.fi
Jaak Viitso, jaak.viitso@tyks.fi
