# Supplementary figures and images for: Comparative analysis of patient-reported outcomes in joint arthroplasty surgeries
Source: PLoS One. 2024 Dec 23;19(12):e0314818. doi: 10.1371/journal.pone.0314818 (PMC11666041; doi:10.1371/journal.pone.0314818)

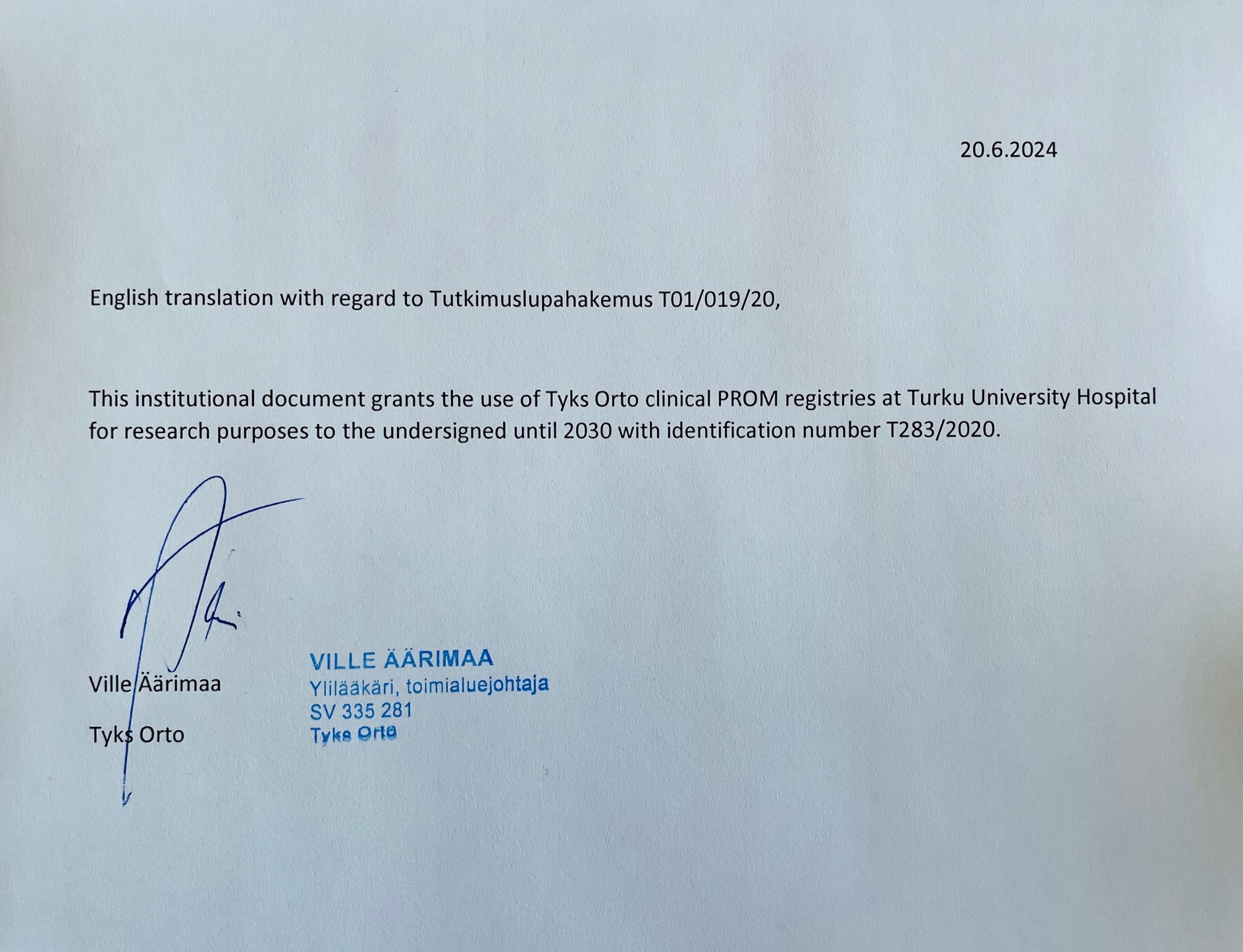

Supplement: S2 File — (JPEG) [file pone.0314818.s002.jpeg]
