# Supplementary material for: Comparative analysis of patient-reported outcomes in joint arthroplasty surgeries
Source: PLoS One. 2024 Dec 23;19(12):e0314818. doi: 10.1371/journal.pone.0314818 (PMC11666041; doi:10.1371/journal.pone.0314818)
Supplement: S1 Table — Note that both numbers refer to the entire registry and thus the operation percentages do not refer only to the diagnosis on the same row of the table. (DOCX) [file pone.0314818.s003.docx]

**Supplementary table 1**

|  | **Diagnosis** |  |  | **Operation** |  |
| --- | --- | --- | --- | --- | --- |
| Shoulder (WOOS) | M19.0 | 92% |  | NBB20 | 99% |
|  | M19.1 | 6% |  | NBU00 | 1% |
|  | M19.2 | 2% |  |  |  |
| Hip (OHS) | M16.1 | 55% |  | NFB40 | 69% |
|  | M16.0 | 39% |  | NFB30 | 28% |
|  | M16.3 | 2% |  | NFB50 | 2% |
| Knee (KOOS) | M17.1 | 52% |  | NGB20 | 91% |
|  | M17.0 | 45% |  | NGB10 | 5% |
|  | M17.3 | 2% |  | NGB40 | 3% |
