# Supplementary material for: Comparative analysis of patient-reported outcomes in joint arthroplasty surgeries
Source: PLoS One. 2024 Dec 23;19(12):e0314818. doi: 10.1371/journal.pone.0314818 (PMC11666041; doi:10.1371/journal.pone.0314818)
Supplement: S2 Table — (DOCX) [file pone.0314818.s004.docx]

**Supplementary table 2**

| **Variable** | **Estimate** | **SE** | **p-value** |
| --- | --- | --- | --- |
| Intercept ^(a^ | 6.34 | 0.885 | <0.0001 |
| Age_65_ ^(b^ | -0.04 | 0.017 | 0.02 |
| Preop. PROM (%) | -0.057 | 0.007 | <0.0001 |
| ASA |  |  |  |
| II | -0.361 | 0.451 | 0.42 |
| III | -0.413 | 0.467 | 0.38 |
| IV | -1.733 | 1.187 | 0.14 |
| Hip | 0.623 | 0.77 | 0.41 |
| Knee | 0.252 | 0.749 | 0.74 |

^(a^ Baselevel is “shoulder”, age 65, ASA class I

^(b^ zero for ages 65 and below, Age–65 for older patients
