# Supplementary material for: Comparative analysis of patient-reported outcomes in joint arthroplasty surgeries
Source: PLoS One. 2024 Dec 23;19(12):e0314818. doi: 10.1371/journal.pone.0314818 (PMC11666041; doi:10.1371/journal.pone.0314818)
Supplement: S3 Table — (DOCX) [file pone.0314818.s005.docx]

**Supplementary table 3**

| **Variable** | **Estimate** | **SE** | **p-value** |
| --- | --- | --- | --- |
| Intercept ^(a^ | 84.00 | 1.99 | <0.0001 |
| Age_65_ ^(b^ | -0.16 | 0.057 | 0.005 |
| Preop. PROM (%) | -0.79 | 0.02 | <0.0001 |
| ASA |  |  |  |
| II | -4.05 | 1.18 | 0.0006 |
| III | -6.22 | 1.26 | <0.0001 |
| IV | -10.67 | 4.99 | 0.03 |
| Hip | 0.70 | 1.69 | 0.68 |
| Knee | -5.76 | 1.68 | 0.0006 |

^(a^ Baselevel is “shoulder”, age 65, ASA class I

^(b^ zero for ages 65 and below, Age–65 for older patients
